# The role of worldviews in the governance of sustainable mobility

**DOI:** 10.1073/pnas.1916936117

**Published:** 2020-02-07

**Authors:** Frank Chuang, Ed Manley, Arthur Petersen

**Affiliations:** ^a^Bartlett Centre for Advanced Spatial Analysis, University College London, London WC1E 6BT, United Kingdom;; ^b^Department of Science, Technology, Engineering and Public Policy, University College London, London WC1E 6BT, United Kingdom;; ^c^School of Geography, University of Leeds, Leeds LS2 9JT, United Kingdom

**Keywords:** worldview, sustainable mobility, culture, cognition, governance

## Abstract

Sustainable development aims for a viable interaction between human and physical nature. However, how do we perceive the social and natural world, rationalize our behavior, and modify our ways of life? Here, we apply the idea of worldviews to cognition and rationality in transport since a transition to sustainable mobility is crucial in dealing with global climate change. We utilize Cultural Theory and the British Social Attitudes survey (*N* = 1,120) to study how three worldviews—egalitarianism, hierarchy, and individualism—relate to people’s attitudes to sustainable mobility. First, we use factor analysis to extract the three worldviews or ways of life in Great Britain. Second, we construct hypotheses concerning the correlations between the worldviews and social attitudes to sustainable mobility. Our statistical analysis of 11 mobility issues in the survey confirms our hypotheses, elucidating the cultural cognition or rationality that underlies people’s transport decision-making. Egalitarianism favors demand control, environmental friendliness, and action driven by inner conviction; hierarchy privileges conformity, order, and security; and individualism embraces freedom, speed, and external incentives. The findings show that the worldviews have a systematic and comprehensive impact on how people assess sustainable mobility debates. Moreover, we perform regression analysis to investigate how these cultural styles are associated with British people’s sociodemographics and political party identification, which can help identify the characteristics of stakeholders in sustainability planning and engagement. We conclude that the worldviews form the bedrock of individual decisions on sustainable mobility and have a wider significance for holistic sustainability governance.

## Significance

In sustainability policy-making, a critical task is to value present and future needs in order to realize good quality of life. To analyze complex ideas of how people interpret reality, develop value orientations, and define needs and the good life, the notion of worldviews proved to be useful. We use worldviews to study how people of distinct ways of life perceive and assess sustainable mobility issues. Through exploring three worldviews (egalitarianism, hierarchy, and individualism), our results map across British people’s attitudes to mobility debates in terms of the economic, environmental, social, and political dimensions. In so doing, our study demonstrates a framework for identifying what behavioral and institutional barriers hinder the transformations needed to achieve better cities and societies.

Transport enables us to fulfill social interaction and to access education, jobs, goods, and a wide range of services that underpin our daily lives. However, the transport sector places a huge burden on our environment and societies. Currently, it contributes to approximately one-quarter of global energy-related greenhouse gas emissions and more than 1.2 million people killed annually in road accidents (1). A transition to sustainable mobility has been urgently needed and high on policy and political agendas in many countries. Like many other sustainable development issues, mobility is frequently a battlefield of value conflicts across the economic, environmental, social, and political domains (2–4). For example, building a motor highway to divert traffic may cause degradation of green fields or demolition of cultural heritage, and implementing a road pricing policy to deter car use may hinder urban and regional economies and damage the rights of the poor and those in situations of inaccessibility.

Addressing the problem of sustainable mobility or contemporary sustainability issues requires a strategy of postnormal science (5): that is, in addition to “hard,” objective scientific facts, “soft,” subjective value judgments have to be integrated into sustainability assessments and decision-making (6–8). This is because different people have distinct needs, definitions of good quality of life, and expectations of the future (8, 9). Moreover, the Brundtland Report states that sustainable development is to “meet the needs of the present without compromising the ability of future generations to meet their own needs” (10). Hence, a central question of sustainable development involves how to value present and future needs in order to realize good quality of life. To analyze complex ideas of how people interpret reality, develop value orientations, and define needs and the good life, the notion of “worldviews” has often been utilized. Worldviews shape people’s visions and mental models, lying at the root of human behavior (11, 12). Therefore, one’s worldview has a profound influence on his/her behavior and societal sustainability (8, 9, 13, 14).

This study aims to unravel the role of worldviews in the governance of sustainable mobility. We attempt to answer some key questions. For example, how do people perceive the interaction between the social and the natural worlds? How can this perception have an impact on their attitudes to urban environmental and transport issues? What are the possible solutions to sustainable mobility planning and urban governance in general? Here, we measure the prevalence of different worldviews in Great Britain using Cultural Theory (which provides a parsimonious model for understanding worldviews) and the British Social Attitudes (BSA) survey (15). Then, the relationships between the worldviews and British social attitudes to sustainable mobility are investigated. Following that, these attitudes are compared according to our grouping of worldview adherents. Finally, through integrating Cultural Theory with policy studies, we argue for the need to adopt an approach of “clumsy solutions,” which are solutions that appeal for combinations of different worldviews, as an effective governance framework for sustainable mobility or development in general.

## Worldviews and Sustainability

**Fig. 1.**
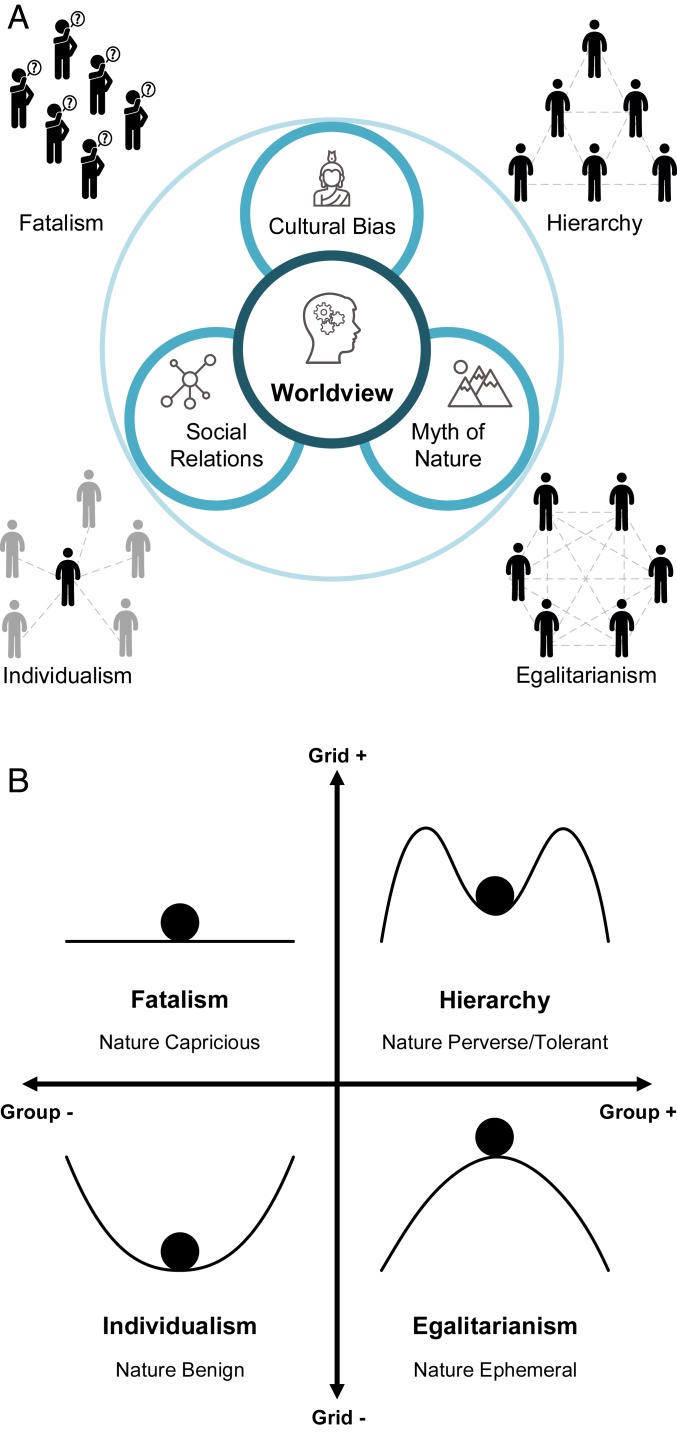
Worldviews or cultural maps: four ways of life, political cultures, or rationalities. (*A*) A worldview is a combination of cultural bias, social relations, and a myth of nature. (*B*) Typology of social relations (group and grid) and myths of nature (adapted with permission from ref. 26).

The term worldview is widely used in the social sciences. It is also related to ideology in politics and cosmology in philosophy or the natural sciences. A worldview is the fundamental cognitive orientation of a person or group regarding the world and life—how people make sense of human and physical nature (6, 8, 9, 16, 17). It encompasses a set of values and beliefs, which “influences such things as how we see ourselves as individuals, how we interpret our role in society, how we deal with social issues, and what we regard as truth” (18). In the literature, Cultural Theory (16, 17, 19–24) is a particular influential body of theoretical and empirical discourses on worldviews. It is a social theory pioneered by Mary Douglas, a British social anthropologist generally considered a follower of Émile Durkheim. Yet, the theory’s influence has spanned a multitude of disciplines and impactfully been formulated in “systems thinking” terms. The theory postulates that a worldview is a combination of cultural bias, social relations, and a myth of nature (17). Here, cultural bias refers to shared values and beliefs, and social relations refer to social organizational forms, while a myth of nature (17, 25–28) is a perspective on the natural world. As illustrated in Fig. 1*A*, the three elements are justifying and reinforcing each other, generating four socially viable worldviews or ways of life: egalitarianism, hierarchy, individualism, and fatalism.

According to the theory, all societies or individuals can be classified into the four worldviews by the two dimensions of social relations: group (social integration) and grid (social regulation) as depicted in Fig. 1*B*. The group dimension specifies how strong group boundaries are or the extent to which individuals’ behavior is constrained by group determination. The grid dimension defines how strong binding prescriptions are or the extent to which individuals’ behavior is constrained by status or role differentiation. Each worldview represents the way that its adherents organize, perceive, and justify social relations, constructing a bias about the interaction between the social and the natural worlds and guiding their thought and action (29). It is a belief system that shapes the adherents’ perceptions, attitudes, values, and preferences. As social and political life demands organization and mobilization of bias (20), the theory also maintains that people’s political attitudes and preferences are derived from the worldviews, which are referred to as political cultures (17, 22, 23).

In an egalitarian social setting, actors’ incorporation within their social units is strong, whereas subordination to binding prescriptions is weak (high group, low grid). The actors have tight connections but tend to reject excess social stratification as they worry that it would bring about corruption and inequality. To satisfy their desire to transform society in an egalitarian direction, their myth of nature has to justify a view that nature is ephemeral and fragile—nature is like a ball on top of a mound, and a slight interference in it would lead to catastrophic consequences (17). Natural or any other resources should not be exploited relentlessly in order to preserve intra- and intergenerational equity. Therefore, environmental concern is rooted in an egalitarian culture (20).

By contrast, a hierarchical culture is characterized by strong social incorporation and prescriptions (high group, high grid). Actors in this context adopt a collective way of life established by authority and role differentiation. Social conformity, order, security, government, expert knowledge, and regulations are respected. This culture generally holds nature to be perverse/tolerant—nature is portrayed as a ball that can roll within limits and come back to the center safely, and government is responsible for managing the boundary line between the normal and abnormal states of nature (17).

In an individualist social setting, both group boundaries and binding prescriptions are condemned and should be negotiated (low group, low grid). Individual freedom is a high priority in this culture, and actors here are self-seeking and self-reliant (17). They endorse competition, entrepreneurship, and free markets to fulfill their individual success. They often embrace a view that nature is robust to manmade turmoil and thus, benign, just as a ball can always return to equilibrium however far it is pushed away (17). This worldview is rationalized by the belief that innovations can break the limits to growth and support economic and social prosperity.

Lastly, fatalism features low social incorporation but strong prescriptions (low group, high grid). Actors in a fatalist context are isolated or alienated from their social units and excessively regulated. Prisoners or the very poor might be in these circumstances. The actors have little choice about decisions for their life. Cooperation with others or political participation is of no use to them (29). This way of life often adopts a myth of nature that sees nature as capricious—the world is so random or unknown that you cannot predict where the ball would go (17).

Cultural Theory promotes an idea of cultural/institutional cognition (21) that brings together social practices, political processes, human life, and sustainable development. It seeks to overcome the dualisms between institutions and individuals and between human and physical nature (17). It has been used for analyzing the opposing views on a wide range of sustainability issues, including climate change policy (29), perceived climate change risks (30), environmentalism (31), renewable energy policy (32), support for nuclear power (33), car users’ attitudes (34), water risk management (35), and global sustainability modeling (36) to name but a few. The theory proved to be useful for investigating risk perceptions through unraveling “who fears what and why” (37). The theory and its accompanying empirical research have proposed that egalitarianism entails stronger perception of technological and environmental dangers than do hierarchy and individualism. For example, Douglas and Wildavsky (20) in their case study of American environmental history compare environmental groups in the United States and argue that a more hierarchical one (e.g., the Sierra Club) is prepared to make compromises between economic demands and environmental conservation, whereas a more egalitarian one (e.g., Friends of the Earth and the antinuclear Clamshell Alliance) tends to highlight potentially disastrous environmental and social consequences (20). However, this does not mean that egalitarianism is always more risk-averse than hierarchy and individualism. As cultural theorists indicate with empirical support (37), egalitarians view technological and environmental hazards as greater in that they believe that insulting nature is just like exploiting the poor; hierarchists deem acts of social deviance more dangerous in that such behavior may undermine social norms that they wish to defend; and individualists perceive more threat of war, which may disrupt markets and individual autonomy. To support their ways of life, organizations’ or individuals’ risk perception is selective and biased, varying with the object of concern (20, 37). People of distinct worldviews have different ranking of risks and preferences (20, 22, 37). In sum, the definition of sustainability differs among worldviews.

## Worldviews and Sustainable Mobility

Built on Cultural Theory, some narratives about mobility in terms of different ways of life have been framed. As noted by Hendriks (2, 38) as well as Hoppe and Grin (39), egalitarians tend to see urban transport problems as demand-side issues. To make cities more livable and sustainable, they believe that reducing the demand for mobility or car use is necessary. They prefer to change transport behavior through inner conviction, such as education, discussion, and good examples. Urban policy promoting diverse means of transport is endorsed by them because this satisfies their desire to transform cities into being more equitable. By contrast, hierarchists in general favor supply-side strategies for dealing with transport problems. For them, infrastructural expansion should not be problematic as long as it does not cause chaos in cities. Traffic guidance systems, rules, and technocracy are preferred to keep cities orderly, safe, controllable, and predictable. Traditional city or transport engineers are prone to accept this culture. Lastly, individualists also tend to define transport problems in terms of supply. The external or environmental costs incurred by car use are not a serious concern. Automobility is to fulfill their freedom of travel, and any barrier to this should be eliminated. Whether they switch to sustainable transport is mainly driven by external incentives. According to Cultural Theory and these associated “mobility narratives,” we developed some hypotheses regarding sustainable mobility.

### Hypothesis 1.

As egalitarianism places the most value on environmental sustainability, embodies demand control, and signifies action driven by inner conviction, it should correlate positively with mobility concerns pertaining to the natural environment, such as the support for reducing car use, the approval of proenvironmental transport policies, the perception of air pollution, and the willingness to switch to low-carbon cars. On the contrary, individualism should be negatively related to these issues. Hierarchy should have an ambiguous correlation with them because the attitudes of a hierarchical culture hinge highly on how technocracy manages the boundary lines between the dilemmas of sustainable urban development as illustrated earlier by the ball rolling in the valley.

### Hypothesis 2.

Because a hierarchical culture privileges conformity, order, and security, hierarchy should correlate positively with mobility concerns linked with social acceptance of transport means and obedience to traffic rules.

### Hypothesis 3.

Since individualism embraces competition and freedom, it should correlate positively with the concern about traffic congestion, an intolerant problem to individualists that endangers their valuable time and opportunities. Also, hierarchy should correlate positively with this concern because congestion symbolizes urban disorder.

It should be noted that we did not include fatalism in our analysis, mainly because previous research concluded that fatalism was inconsequential in understanding environmental attitudes and frequently excluded it from policy analysis (31, 36, 40). Before testing our hypotheses, worldviews need to be measured, and individuals need to be categorized, which now we will turn to.

## Measurement of Worldviews

**Fig. 2.**
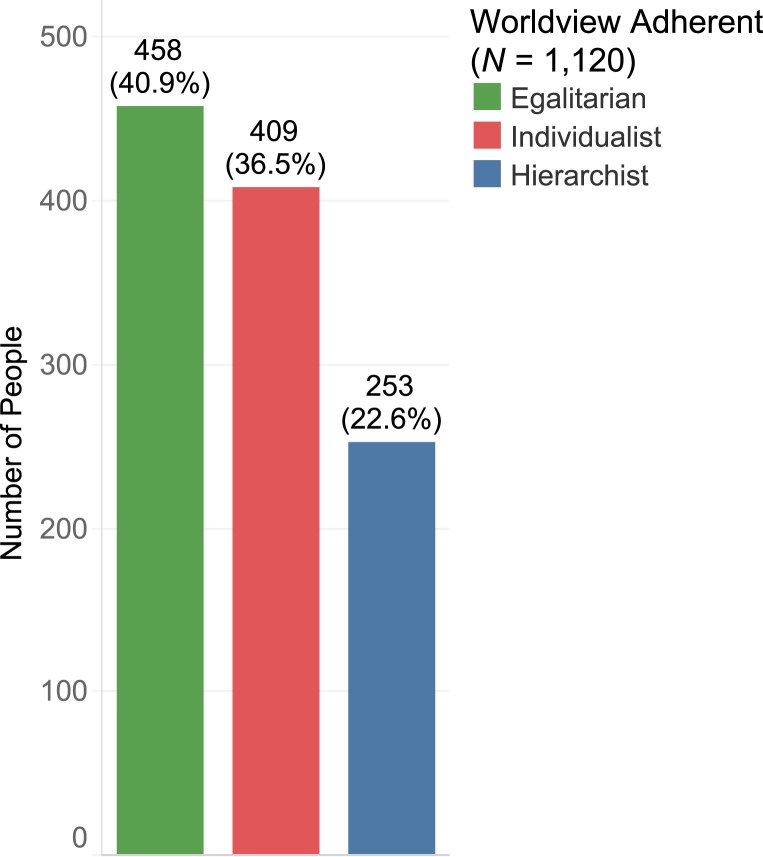
Estimated configuration of worldview adherents in Great Britain (adults aged 18 or over).

Our analysis of the British worldviews and social attitudes to sustainable mobility was carried out on the BSA 2016 (15). The BSA is a large-scale, annual government survey conducted in Great Britain by the National Centre for Social Research since 1983. The survey is designed to produce a representative sample of adults aged 18 or over. As an important barometer of public opinions to inform public policy, it covers a whole range of topics, such as welfare, health, education, gender, employment, transport, the environment, and political parties. Based on our factor analysis (*N* = 1, 120) of the dataset, Fig. 2 depicts our estimated proportions of the three types of worldview adherents in the British society. As shown here, the society was dominated by egalitarians followed by individualists and hierarchists. In the remainder of this section, we explain how we arrived at this result.

Measuring the worldviews defined by Cultural Theory can be performed in various ways. One of the earliest approaches is to measure social relations (group and grid) at the organizational level (41). However, this needs to rely on in-depth ethnographic studies and thus, may not be suitable for large-scale circumstances (41). Therefore, worldviews are also estimated using survey data at the individual level: for example, through the lens of (political) cultural bias (31, 42–44), social relations (45, 46), or myths of nature (34, 47). Since cultural bias and social relations are the core of the theory, we preferred to choose one between the two. As the BSA survey is not primarily intended for capturing notions of social relations or network structure, we finally determined to measure the British worldviews by analyzing (political) cultural biases at the individual level.

Political cultures, which embody preferences for policies and institutions, are “the social filter enabling people who possess only inches of facts to generate miles of preferences” (22). In other words, after people opt for a particular institutional setting, their attitudes and behaviors can be inferred by that choice (31). Accordingly, we identified nine BSA survey items that parallel the political cultures defined by Cultural Theory as listed in Table 1. Each response related to the level of agreement with a proposed statement. Originally, eight of the nine items were rated on a five-point Likert-type scale ranging from one to five (agree strongly, agree, neither agree nor disagree, disagree, disagree strongly, respectively), while only the third survey item was rated on a four-point Likert-type scale ranging from one to four (definitely should be, probably should be, probably should not be, definitely should not be, respectively). For ease of interpretation, all of their directions were reversed before our analysis (e.g., one for disagree strongly and five for agree strongly).

**Table 1.** Factor loadings of survey items on worldviews

	Factor (proposed worldview)		
Survey item	Egalitarianism	Hierarchy	Individualism
1. Government should redistribute income from the better-off to those who are less well-off.	0.73*	–0.13	
2. There is one law for the rich and one for the poor.	0.64*		
3. Government should reduce income differences between the rich and the poor.	0.60*		–0.15
4. Schools should teach children to obey authority.		0.70*	
5. Young people today do not have enough respect for traditional British values.		0.60*	0.10
6. Censorship of films and magazines is necessary to uphold moral standards.		0.54*	
7. Government should not spend more on unemployment benefits.		–0.10	0.79*
8. If welfare benefits were not so generous, people would learn to stand on their own two feet.		0.30	0.62*
9. Cutting welfare benefits would not damage too many people’s lives.	–0.13		0.61*

*N* = 1,120. The third item was rated on a four-point scale, while the others were rated on a five-point scale. Factors were rotated with the oblique (Direct Oblimin) method with Kaiser normalization. Only factor loadings with absolute values no smaller than 0.10 are reported. The direction of the ninth survey question was reversed. The original statement was as follows: cutting welfare benefits would damage too many people’s lives.

* The factor loadings of practical significance.

The proportions of the three types of worldview adherents in Great Britain were estimated using factor analysis, a dimension reduction technique for scientific research. It can extract the underlying factors, or latent constructs, of a set of observed variables so that researchers can better interpret their data or justify a theory. It is usually used to reduce information or a dataset to a more manageable number of dimensions, or factors, through identifying variables that correlate highly with each other within a group but badly with variables outside that group. The results in Table 1 identified three worldview or political cultural dimensions. Each dimension, or factor, was associated with three items with factor loadings that were greater than 0.50 (i.e., with significance). A factor loading is conceptually a survey item’s contribution to a factor. The higher the loading, the more the factor is accounted for by that item. A rule of thumb suggests that a loading (absolute value) ≥ 0.50 is considered practically significant (48). Furthermore, no item loaded at 0.32 or higher on two or more factors: that is, no cross-loading was found, which means that the three factors (latent constructs) are sufficiently distinct. While there are no strict thresholds for determining a cross-loading, a rule of thumb suggests that an item with more than one loading (absolute value) ≥ 0.32 is deemed a cross-loading, which indicates potential nonseparation of factors (49). Our factor loadings meet the criteria for significance and separation. Further reliability checks of the model are in *Materials and Methods* and *SI Appendix*, Table S3.

The first dimension was composed of the notions concerning redistribution of income, legal equality, and elimination of income differences, which represent the egalitarian cause of equality. Thus, this group was labeled as egalitarianism. The second dimension was characterized by the ideas about obedience to authority, respect for traditional British values, and censorship of media, which portray the hierarchical faith in social conformity. Hence, we labeled it as hierarchy. The third dimension consisted of the items regarding restraint on unemployment benefits, self-reliance, and cut in welfare benefits. Since those ideas describe the individualist norm of competition and self-seeking, this group was labeled as individualism.

For each individual, his/her factor scores for the three worldviews were computed. A factor score is a linear combination of observed variables (the nine BSA political cultural items). It can be used as a composite indicator representing the degree to which an individual scores high on a factor. Factor scores are standardized scores (similar to *z* scores) with a mean of zero. In our analysis, an individual’s factor scores for egalitarianism, hierarchy, and individualism were calculated with Thurstone’s regression method (50), a widely used technique for estimating latent constructs. Then, each individual was categorized as an adherent of a worldview that had his/her highest factor score. Using this procedure, the proportions of the three types of worldview adherents, egalitarians, hierarchists, and individualists, were derived (Fig. 2). Furthermore, the relationships between the three worldview factors and sociodemographics/political party identification were examined. Linear regressions were performed with each worldview factor score as the outcome and sociodemographics/party identification as the predictors. Our results showed tendencies that egalitarianism was associated with people who had a lower household income and identified themselves with non-Conservative parties, including the Labour, Scottish National, and Green Parties; that hierarchy was associated with people who had a lower level of education and identified themselves with not the Labour, Liberal Democrats, Scottish National, or Green Parties; and that individualism was associated with younger males who had a higher household income but a lower level of education, and identified themselves with the Conservative Party (*SI Appendix*, Table S1 has details). This information would be useful for identifying the characteristics of policy stakeholders in sustainability planning and engagement.

## Relationships between Worldviews and Social Attitudes to Sustainable Mobility

We identified 11 BSA survey items concerning attitudes to sustainable mobility issues and investigated their Pearson’s correlations with the three worldviews as shown in Table 2. Each response revealed the level of agreement with a statement. The first nine statements were asked to all survey participants, while the last two were dedicated to car users. Issues 1 to 5, 10, and 11 relate to environmental concerns (Hypothesis 1), including the support for reducing/allowing car use, the approval of proenvironmental transport policies, the perception of air pollution, and the willingness to switch to low-carbon cars; issues 6 to 8 relate to conformity, order, and security (Hypothesis 2); and issue 9 is the concern about traffic congestion (Hypothesis 3). Originally, 9 of the 11 items were rated on a five-point Likert-type scale ranging from one to five (agree strongly, agree, neither agree nor disagree, disagree, disagree strongly, respectively), while the other 2 items (issues 5 and 9) were rated on a four-point Likert-type scale ranging from one to four (a very serious problem, a serious problem, not a very serious problem, not a problem at all, respectively). For ease of interpretation, all of their directions were reversed before our analysis (e.g., one for disagree strongly and five for agree strongly).

**Table 2.** Correlations between worldview factor scores and social attitudes to sustainable mobility

	Egalitarianism		Hierarchy		Individualism	
Issue	Sign	*r*	Sign	*r*	Sign	*r*
Environmental concern and policy (Hypothesis 1)						
1) Reduce car use	⊕	0.15***		–0.08	⊖	–0.12**
2) Allow car use	⊖	–0.25***	⊕	0.18***	⊕	0.25***
3) Higher car tax	⊕	0.26***	⊖	–0.23***	⊖	–0.26***
4) Road price incentive	⊕	0.11**	⊖	–0.08*	⊖	–0.09*
5) Fumes problem	⊕	0.09**		–0.05	⊖	–0.08**
Conformity, order, and security (Hypothesis 2)						
6) Unless others do		–0.01	⊕	0.26***	⊕	0.16***
7) Obey speed limit		0.08	⊕	0.17***		–0.02
8) Bike danger	⊕	0.10**	⊕	0.15***		–0.01
Urban order and flexibility (Hypothesis 3)						
9) Congestion problem		–0.02	⊕	0.07*	⊕	0.08**
Environmental concern (car users only; Hypothesis 1)						
10) Reduce car travel	⊕	0.11**	⊖	–0.11**	⊖	–0.13***
11) Low-carbon car	⊕	0.15***	⊖	–0.08*	⊖	–0.13***

Pearson’s correlation is denoted by *r*. ***P < 0.001 (two-tailed); **P < 0.01 (two-tailed); *P < 0.05 (two-tailed). Issues 1 to 9 were for all survey participants, while issues 10 and 11 were for car users only. Because of the design of the BSA 2016, the sample sizes could vary: 1) issues 1 to 4 and 6: *n* = 572; 2) issues 5, 8, and 9: *n* = 1,120; 3) issue 7: *n* = 548; and 4) issues 10 and 11: *n* = 840. Issues are as follows. 1) Reduce car use: For the sake of the environment, everyone should reduce how much they use their cars. 2) Allow car use: People should be allowed to use their cars as much as they like, even if it causes damage to the environment. 3) Higher car tax: For the sake of the environment, car users should pay higher taxes. 4) Road price incentive: People who drive cars that are better for the environment should pay less to use the roads than people whose cars are more harmful to the environment. 5) Fumes problem: How serious a problem for you are exhaust fumes from traffic in towns and cities? 6) Unless others do: There is no point in reducing my car use to help the environment unless others do the same. 7) Bike danger: It is too dangerous for me to cycle on the roads. 8) Obey speed limit: People should drive within the speed limit. 9) Congestion problem: How serious a problem for you is traffic congestion in towns and cities? 10) Reduce car travel: I am willing to reduce the amount I travel by car to help reduce the impact of climate change. 11) Low-carbon car: Next time I buy a car, I would be willing to buy a car with lower CO_2_ emissions. This might be an ordinary car with a smaller or more efficient engine or a vehicle that runs on electric or alternative fuels.

As predicted by Hypothesis 1, egalitarianism correlated positively with issues 1) reduce car use, 3) higher car tax, 4) road price incentive, 5) fumes problem, 10) reduce car travel, and 11) low-carbon car and negatively with issue 2) allow car use. On the contrary, individualism showed a reverse pattern. Therefore, egalitarianism and individualism revealed two obviously opposing views on the issues of Hypothesis 1. For all of these environmental issues, hierarchy’s directions were consistent with those of individualism, but its magnitudes were all smaller than those of individualism, and its correlations regarding issues 1 and 5 were nonsignificant (i.e., P≥0.05). This confirmed our idea that hierarchy, compared with the other two cultures, should take a middle or ambiguous position in the face of mobility issues concerning the environment.

The results of issues 6) unless others do, 7) obey speed limit, and 8) bike danger aligned with Hypothesis 2, showing that hierarchy attached the greatest importance to them. From a perspective of Cultural Theory, the hierarchical worldview deemed that cycling was dangerous because it was generally believed to be an uncommon transport mode. Finally, as predicted by Hypothesis 3, individualism and hierarchy were both positively related to issue 9) congestion problem because it is a danger to freedom of travel and to urban order. Although some values in Table 2 are small, this is not uncommon for cultural variables such as worldviews. The point is that worldviews can account for a wide range of cognitive or value orientations concerning sustainable mobility and therefore, have a systematic and comprehensive impact on mobility debates across the economic, environmental, social, and political domains. This confirms the idea that worldviews are a critical factor in holistically changing human behavior toward sustainability (8, 9, 13, 14).

## Comparison of Social Attitudes by Worldviews

We also investigated the differences in the means of the three worldview groups’ attitudes to sustainable mobility. For each group, the means for the attitude scores of the selected 11 mobility issues were calculated. Then, for each issue, we conducted an ANOVA to examine whether significant differences could be found between group means. Welch’s *F* statistic was used in the analysis. If the *F* test was significant (at P<0.05), pairwise comparison (post hoc) tests were carried out to identify the group means that differed significantly (at P<0.05). Further details are in *Materials and Methods* and *SI Appendix*, Table S2.

**Fig. 3.**
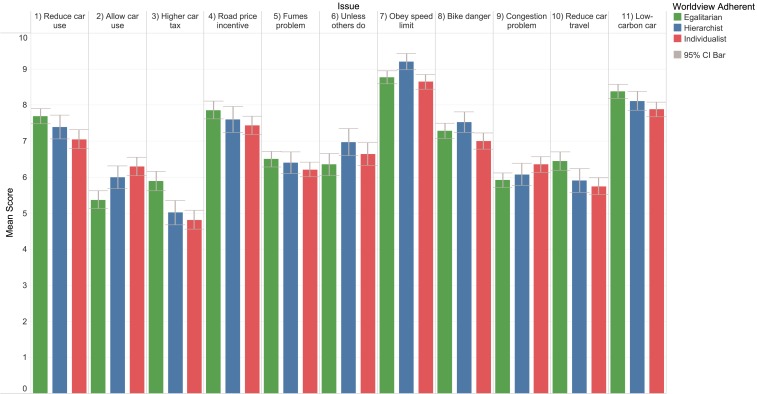
Mean scores of social attitudes to sustainable mobility. Issues 1 to 9 were for all survey participants, while issues 10 and 11 were for car users only. Originally, issues 5 and 9 were rated on a four-point scale, while the others were rated on a five-point scale. The statistical significances of differences in means are reported in *SI Appendix*, Table S2. Because of the design of the BSA 2016, the sample sizes could vary: 1) issues 1 to 4 and 6: *n* = 572 (egalitarian = 242, hierarchist = 123, individualist = 207); 2) issues 5, 8, and 9: *n* = 1,120 (egalitarian = 458, hierarchist = 253, individualist = 409); 3) issue 7: *n* = 548 (egalitarian = 216, hierarchist = 130, individualist = 202); and 4) issues 10 and 11: *n* = 840 (egalitarian = 305, hierarchist = 187, individualist = 348).

The comparison of the British social attitudes is depicted in Fig. 3. For easier visualization and interpretation, the different scales (of 5 or 4 points) used in the BSA were all converted proportionally to a 10-point scale ranging from 1 (low) to 10 (high). The results shown in Fig. 3 paralleled the findings in Table 2. Again, tendencies existed that egalitarians revealed greater concern for the environment: issues 1 to 5, 10, and 11; that hierarchists placed more value on the issues about conformity, order, and security: issues 6 to 8; and that individualists as well as hierarchists attached greater importance to congestion: issue 9. For all of the issues except issues 4 and 5, at least one group mean was significantly different from the others.

Issues 3 and 4 are both in relation to proenvironmental transport policy. Issue 3, imposing higher car taxes, belongs to push policy instruments (penalties), whereas issue 4, implementing a road price incentive, belongs to pull policy instruments (rewards) (51, 52). However, compared with issue 3, which showed a highly significant difference in the group means (P<0.001) (*SI Appendix*, Table S2), the group means for issue 4 had no statistically significant difference (P=0.067) (*SI Appendix*, Table S2). From a psychological perspective, pull policy measures generally elicit less reluctance than do push ones (52). From a viewpoint of Cultural Theory, the polarization among different ways of life can be reduced as a result of the external incentives induced by the pull (or rewarding) policy measure. These effects were so much so that even individualists could not be statistically significantly differentiated from egalitarians in terms of the mean attitude to the measure.

Regarding issue 5, the differences between the group means were nonsignificant (P=0.159) (*SI Appendix*, Table S2). This implies that fumes were not just a problem to one particular way of life; instead, they were perceived as hazardous almost equally by the three groups of worldview adherents. From an egalitarian perspective, fumes are anthropogenic harm to the environment and should be eliminated; for hierarchists, air pollution is highlighted by the government and therefore, considered a serious social issue; and from an individualist angle, breathing the exhaust jeopardizes personal health and freedom of travel.

## Discussion

People use a limited number of heuristic rules to deal with uncertainties and make judgments when they navigate in the complex world (22, 53–55). This study unravels the worldviews—cultural cognition, bias, and rationality—as the heuristic principles for individual decision-making regarding sustainable mobility. However, we need to highlight that we do not suggest that any worldview is superior or can survive on its own. In fact, while the worldviews are rivals, they also need to rely on each other to correct their blind spots and make societies sustainable. Egalitarians help to promote equality, social cohesion, and environmental stewardship; hierarchists help to enforce order, authority, and contracts; and individualists help to advance economic and technological progress through their creative energy (17, 29, 56). Similarly, all of the cultural styles are essential for sustainable mobility. As suggested by our study, the egalitarian way of life favors demand control, environmental friendliness, and action guided by inner conviction; the hierarchical cultural style privileges conformity, order, and security; and the individualist norm embraces freedom, speed, and external incentives. However, if any of the cultures is dropped from an urban system or policy, mobility will not be viable or truly sustainable. The ways of life compete with but ultimately, depend on each other—the requisite variety condition set out in Cultural Theory—suggesting that policy-makers would benefit from accounting for all of the worldviews in their decisions (17).

**Fig. 4.**
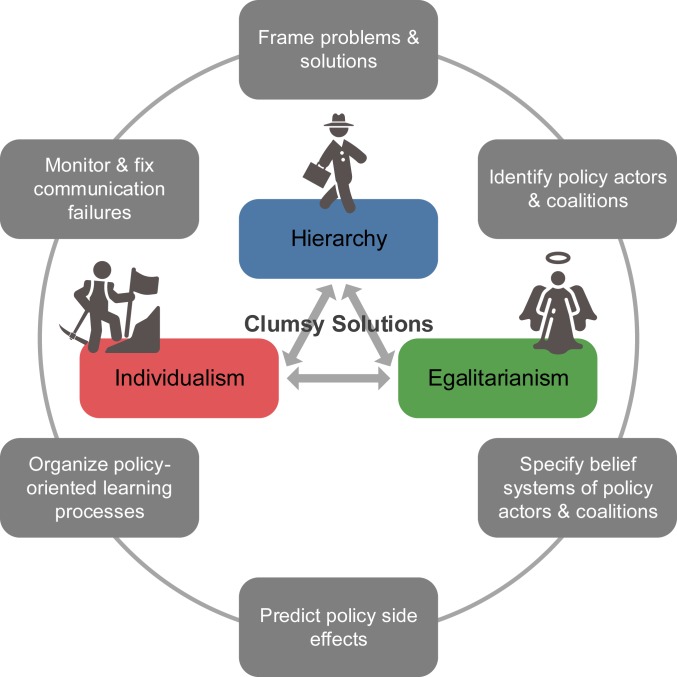
Clumsy solutions for governing sustainable development.

A French metaphor by Christian Brunner (57) portrayed the heroes of the three worldviews as the Holyman with a halo (egalitarianism), the Bureaucrat with a briefcase (hierarchy), and the Pioneer with a pickax (individualism). Because they have distinct ways of life, opposing values and beliefs, and diverse strategies for problem selection and solving, their communication is often like the dialogue of the deaf (24, 57). However, in the face of sustainable development, “elegant solutions,” which adopt the single problem–single solution approach to optimizing the goal of one particular worldview, are no longer satisfactory. To address “wicked problems” (which literally all sustainability issues belong to) involving multiple rationalities, cultural theorists propose clumsy solutions (or called plural rationalities)—all voices should be heard and responded to by the others (29, 58–60). Clumsiness is preferable to elegance after we realize that arguments are actually based on different premises (belief systems) or rival worldviews (58). Through integrating the notion of clumsy solutions with pertinent policy studies (61–65), a “clumsy” governance framework is summarized in Fig. 4, illustrating a variety of functions that clumsiness can perform in the development of sustainability policies.

The value of the framework can be elucidated by the comparative studies of Hendriks (2, 38) on the experience in two major postwar European cities—one was a great success, while the other was full of regrets. After World War II, Birmingham witnessed a rapid growth of cars, and its city engineer proposed the Inner Ring Road (IRR) to support the increasing volume of traffic. This project was endorsed by the city’s policy culture, which was predominated by a strong coalition of hierarchists and individualists. They held consensual policy beliefs in automobility and contributed to the project funding, but they entrapped the city in a single problem–single solution approach that overlooked alternative solutions such as public transport. The IRR was opened in 1971, but its monofunctionality soon proved to be a physical and psychological barrier of the city, and numerous traditional buildings were demolished due to the construction. Many communication failures could have been fixed.

On the other hand, Munich, stimulated by the 1972 Olympic Games, was keen to show the world a revived city. The city’s hierarchists and individualists were particularly enthusiastic about new construction plans, motivating the conception of the Altstadtring Road. However, to preserve cultural heritage, egalitarian organizations popped in, fiercely opposing this project and inspiring more and more initiatives. In such a climate, in 1968 the city mayor invited a group of critical architects and town planners to launch the Münchner Forum as a policy broker to facilitate communication and negotiation between all of the policy actors. Owing to this deliberative institution, the city’s planning proposals were refined continuously through a policy-learning loop based on a multiple problem–multiple solution regime. Ultimately, the Olympic Games opened and showed the world a city that allowed cultural vitality, colorful pedestrian zones, efficient public transport, and the Altstadtring to coexist together. As suggested here, the clumsy solutions, which made the city accessible, livable, and sustainable, did not emerge until policy actors of all of the worldviews joined the three-cornered policy arena.

Worldviews embody our awareness of the social and natural world, which in turn, reflects how we define good quality of life and link our present to the future that we envision. We have demonstrated that worldviews in fact transcend the boundary between human and physical nature and have the potential to map across social attitudes to sustainable mobility. Combined with the idea of clumsy solutions, worldviews also have a wider significance for sustainability governance. Last but not least, it is worthwhile to mention that we have not laid out the fatalist attitudes to sustainable mobility for the sake of simplicity as mentioned earlier, but this does not suggest that fatalist actors should always be ignored. Throughout human history, they are usually the most powerless and exploited, potentially serving as the lower ranks for hierarchists to legitimize social differentiation, as the members to mirror the success of individualists, and time after time, as the followers of egalitarian movements and appeals for equality (8, 17). The complex dynamics of the world are rooted in the competition and cooperation of our worldviews. Whether it is to inform public deliberations, conceive inclusive solutions, or foster paradigm shifts needed to achieve a better common future, worldviews should have a significant role.

## Materials and Methods

The dataset that supports our findings is the BSA 2016 (15). It is designed to yield a representative sample of adults aged 18 or over in Great Britain. It covers public attitudes across the economic, environmental, social, and political domains. The survey is conducted by both the face-to-face interview and self-completion (the latter is usually for particularly sensitive questions). The original sample size of the BSA 2016 is 2,942. The survey has several versions of the questionnaire, and each respondent was asked a randomly selected version. Not all of the versions include the cultural bias items required for our analysis. After excluding the entries lacking cultural bias or sociodemographics, the total number of the individuals that we analyzed is 1,120. Since no nonrandom association between question selection and respondents was identified, we can conclude that no selection bias is introduced at this stage.

The analytical tool that we used to conduct this study is IBM SPSS Statistics 26. The proportions of the three types of worldview adherents in the British society were estimated using factor analysis, a method that can help group similar variables into dimensions or factors. The choice of the number of dimensions can be suggested by factor eigenvalues. A factor’s eigenvalue represents the amount of variance in the original variables accounted for by the factor. The initial exaction of our data revealed that the eigenvalues of two factors were larger than 1.0 (individualism = 3.14, hierarchy = 1.77, and egalitarianism = 0.95). However, the three-factor solution was selected because it revealed no cross-loadings as is explained earlier. The three factors jointly explained 65.11% of the total variance in the original variables. Moreover, the factors in our analysis were rotated using the oblique (Direct Oblimin) method as it can make clearer the factor structure without needing to assume that they are orthogonal (i.e., independent). The rotated results are given earlier in Table 1, which shows that three factors are identified; each of them was contributed mainly by three survey items and labeled as a worldview.

The checking of each factor’s reliability, or intragroup consistency, is documented in *SI Appendix*, Table S3. The reliability statistic (Cronbach’s α) for each group was between 0.65 and 0.76. The item-to-total correlations were all greater than or equal to 0.40. While there are no strict thresholds for these measures, rules of thumb suggest that an α≥ 0.60 is considered acceptable (48) and that an item-to-total correlation ≥ 0.30 is deemed adequate (66). Our factor model meets these criteria. Furthermore, each α value could not be improved by deleting any item in its own group. Therefore, the survey items within each group can be considered to reliably measure the same latent construct.

After the reliability checks, each individual in the data was categorized as an egalitarian, hierarchist, or individualist according to his/her highest worldview factor score. All factor scores were calculated with Thurstone’s regression method (50). After categorizing the individuals, the group means of their attitude scores for each sustainable mobility issue were compared. ANOVA was used to examine whether significant differences could be found between group means (*SI Appendix*, Table S2 has details). Welch’s *F* statistics were used in our analysis to ensure that the results were robust even if the assumption of homogeneity of variance in ANOVA was violated. For those cases in which *F* tests were significant (at P<0.05), post hoc tests were conducted to identify the group means that differed significantly (at P<0.05). Our post hoc tests used the Games–Howell procedure, which is customarily available in standard statistical packages.

### Data Availability.

The dataset supporting the findings of this study is the British Social Attitudes survey 2016, which is available at the UK Data Service (https://doi.org/10.5255/UKDA-SN-8252-1 or https://beta.ukdataservice.ac.uk/datacatalogue/studies/study?id=8252).

## Supplementary Material

Supplementary File
