# Supplementary material for: The role of worldviews in the governance of sustainable mobility
Source: Proc Natl Acad Sci U S A. 2020 Feb 7;117(8):4034–42. doi: 10.1073/pnas.1916936117 (PMC7049160; doi:10.1073/pnas.1916936117)
Supplement: Supplementary File [file pnas.1916936117.sapp.pdf]

Supplementary Information
www.pnas.org/cgi/doi/10.1073/pnas.1916936117

The role of worldviews in the governance of sustainable mobility

Frank Chuang[a,b,1], Ed Manley[a,c], and Arthur Petersen[b]

[a]Bartlett Centre for Advanced Spatial Analysis, University College London, London WC1E 6BT, United Kingdom
[b]Department of Science, Technology, Engineering and Public Policy, University College London, London WC1E 6BT, United Kingdom
[c]School of Geography, University of Leeds, Leeds LS2 9JT, United Kingdom
[1]Email: feng-yuan.chuang.15@ucl.ac.uk

Table S1. Standardized regression coefficients showing the relationships between worldview factor scores and sociodemographics/political party identification

	Egalitarianism	Hierarchy	Individualism
Sociodemographics			
Sex	−0.026	−0.043	−0.070*
Age	0.049	0.035	−0.078**
Household income	−0.196***	−0.040	0.137***
Educational level	−0.021	−0.277***	−0.082**
Political party identification			
Conservative Party	−0.313***	0.019	0.198***
Labour Party	0.145**	−0.192***	−0.215***
Liberal Democrats Party	−0.002	−0.136***	−0.088**
Scottish National Party	0.086**	−0.140***	−0.105***
Green Party	0.084**	−0.156***	−0.132***
UK Independence Party	−0.049	0.025	0.037
Other	−0.024	−0.037	−0.005
N	1,120	1,120	1,120
R^2	0.26	0.19	0.21
R^2_{adj}	0.25	0.18	0.20

- ***$P < 0.001$ (two-tailed); **$P < 0.01$ (two-tailed); *$P < 0.05$ (two-tailed).

- Coding of independent variables:
  1. Sex: *male* (0) and *female* (1).
  2. Age: 7 bands, from *18-24* (1) to *65 or more* (7).
  3. Household income: 20 bands, from *monthly less than £590 before tax* (1) to *monthly £7,201 or more before tax* (20).
  4. Educational level: 7 bands, from *no qualification* (1) to *degree* (6). The *foreign or other* qualification is replaced with the mean educational level (3.64).
  5. Political party identification: 7 dummy variables for the 7 groups of parties. *None* (no party identification) is set as the baseline group.

Table S2. ANOVA and pairwise comparison (*post hoc*) tests comparing the group means of social attitudes to sustainable mobility

Issue		Welch's F statistic	P value from ANOVA	Ranking
1)	Reduce car use	$F(2, 304.20) = 6.85$	$P = 0.001$	$E > I$
2)	Allow car use	$F(2, 312.10) = 13.21$	$P < 0.001$	$E < H, I$
3)	Higher car tax	$F(2, 319.61) = 17.02$	$P < 0.001$	$E > H, I$
4)	Road price incentive	$F(2, 301.40) = 2.73$	$P = 0.067$	–
5)	Fumes problem	$F(2, 628.44) = 1.85$	$P = 0.159$	–
6)	Unless others do	$F(2, 312.65) = 3.16$	$P = 0.044$	$H > E$
7)	Obey speed limit	$F(2, 322.00) = 7.16$	$P < 0.001$	$H > E, I$
8)	Bike danger	$F(2, 647.50) = 4.30$	$P = 0.014$	$H > I$
9)	Congestion problem	$F(2, 626.13) = 3.98$	$P = 0.019$	$I > E$
10)	Reduce car travel	$F(2, 472.67) = 8.37$	$P < 0.001$	$E > H, I$
11)	Low-carbon car	$F(2, 477.27) = 6.41$	$P = 0.002$	$E > I$

- Issues 1 to 9 were for all survey participants, while issues 10 and 11 were for car users only.

- Only statistically significant differences in means (identified by ANOVA and *post hoc* tests, both at $P < 0.05$) are reported in the ranking list.

- Because of the design of the BSA 2016, the sample sizes could vary:
  1. Issues 1 to 4 and 6: $n = 572$ (egalitarian = 242, hierarchist = 123, individualist = 207).
  2. Issues 5, 8, and 9: $n = 1,120$ (egalitarian = 458, hierarchist = 253, individualist = 409).
  3. Issues 7: $n = 548$ (egalitarian = 216, hierarchist = 130, individualist = 202).
  4. Issues 10 and 11: $n = 840$ (egalitarian = 305, hierarchist = 187, individualist = 348).

Table S3. Reliability checks of survey items for factor analysis

Survey item	Item-to-total correlation	Cronbach's α if item deleted
Egalitarianism		
1. Government should redistribute income from the better-off to those who are less well-off.	0.57	0.56
2. There is one law for the rich and one for the poor.	0.47	0.69
3. Government should reduce income differences between the rich and the poor.	0.55	0.60
Cronbach's $\alpha = 0.71$		
Hierarchy		
4. Schools should teach children to obey authority.	0.51	0.47
5. Young people today do not have enough respect for traditional British values.	0.44	0.55
6. Censorship of films and magazines is necessary to uphold moral standards.	0.40	0.62
Cronbach's $\alpha = 0.65$		
Individualism		
7. Government should not spend more on unemployment benefits.	0.60	0.68
8. If welfare benefits were not so generous, people would learn to stand on their own two feet.	0.60	0.68
9. Cutting welfare benefits would not damage too many people's lives.	0.59	0.69
Cronbach's $\alpha = 0.76$		

- $N = 1,120$.
- Cronbach's α is based on standardized items.
